# The effect of sugar and processed food imports on the prevalence of overweight and obesity in 172 countries

**DOI:** 10.1186/s12992-018-0344-y

**Published:** 2018-04-14

**Authors:** Tracy Kuo Lin, Yasmin Teymourian, Maitri Shila Tursini

**Affiliations:** 1 University of California, San Francisco, USA; 2 London School of Economics and Political Science, London, UK; 3 London School of Hygiene and Tropical Medicine & Homerton University Hospital, London, UK

**Keywords:** Globalization, Obesity, Imports, Synthetic control method

## Abstract

**Background:**

Studies find that economic, political, and social globalization – as well as trade liberalization specifically – influence the prevalence of overweight and obesity in countries through increasing the availability and affordability of unhealthful food. However, what are the mechanisms that connect globalization, trade liberalization, and rising average body mass index (BMI)? We suggest that the various sub-components of globalization interact, leading individuals in countries that experience higher levels of globalization to prefer, import, and consume more imported sugar and processed food products than individuals in countries that experience lower levels of globalization.

**Method:**

This study codes the amount of sugar and processed food imports in 172 countries from 1995 to 2010 using the United Nations Comtrade dataset. We employ country-specific fixed effects (FE) models, with robust standard errors, to examine the relationship between sugar and processed foods imports, globalization, and average BMI. To highlight further the relationship between the sugar and processed food import and average BMI, we employ a synthetic control method to calculate a counterfactual average BMI in Fiji.

**Conclusion:**

We find that sugar and processed food imports are part of the explanation to increasing average BMI in countries; after controlling for globalization and general imports and exports, sugar and processed food imports have a statistically and substantively significant effect in increasing average BMI. In the case of Fiji, the increased prevalence of obesity is associated with trade agreements and increased imports of sugar and processed food. The counterfactual estimates suggest that sugar and processed food imports are associated with a 0.5 increase in average BMI in Fiji.

**Electronic supplementary material:**

The online version of this article (10.1186/s12992-018-0344-y) contains supplementary material, which is available to authorized users.

## Background

Trade flows are assumed to be associated with economic growth, which in turn has a positive relationship with the level of living standards and public health [1]. However, while the prevalence of communicable diseases has been reduced and life expectancy has improved substantially in countries with growing economies, these countries also face a spread of non-communicable diseases, such as obesity [2].

Trade – an aspect of economic globalization – is only part of the story explaining the rapid rise in obesity around the world. In addition to economic globalization, studies outline political globalization and social globalization as sub-components of globalization. Each of these sub-components has a unique association with the increased prevalence of overweight and obesity; and together, they stimulate increased calorie consumption and smaller energy expenditure [3].

We suggest that one of the results of the interaction between these sub-components is increased consumption of imported sugar and processed food due to 1) trade liberalization [4, 5] and trade agreements that reduce some governments’ power in regulating unhealthful food [6] and 2) greater preference for Western food [7]. Health economic literature argues that the import of goods, including a large amount of processed food, often accompanies economic liberalization. The availability of processed food and citizens’ ability and preference to purchase them play a crucial role in increasing overweight and obesity rate.

This study contributes to the literature on globalization’s effect on non-communicable diseases by quantitatively evaluating the relationship between globalization, sugar and processed food imports, and the prevalence of overweight and obesity. Utilizing a newly coded dataset on sugar and processed food imports worldwide – derived from the United Nations Comtrade Dataset – we demonstrate that one of the mechanisms through which globalization leads to increased prevalence of overweight and obesity is by stimulating imports of sugar and processed food. We then employ Fiji as an example and include a qualitative description and a quantitative prediction of trends in average body mass index (BMI) to highlight the effect of sugar and processed food imports on average BMI.

The rest of the paper is as follows: First, the paper discusses obesity as a disease and summarizes the literature on globalization, emphasizing trade and health. Next, we evaluate the impact of globalization and sugar and processed food on obesity. Then, we test the hypothesis that sugar and processed food imports are associated with average BMI in countries. Finally, the paper concludes that sugar and processed food imports are specific factors – which accompany globalization and increased trade flow – that are associated with increased average BMI in overweight and obese countries.

### Obesity and disease

Obesity is a disease and is a risk factor for several different pathologies; it is defined as “abnormal or excessive fat accumulation that presents a risk to health” [8] and is quantified with the BMI, which is an index of weight in relation to height. The World Health Organization (WHO) defines a person with BMI that is greater than or equal to 25 as overweight and a person with BMI that is greater than or equal to 30 as obese [8]. It is estimated that more than 1.4 billion people over the age of 20 are overweight worldwide. Moreover, over 200 million of these overweight individuals are obese. The number amounts to about 10% of the world population being obese [9].

The aetiology of obesity is complex and multifactorial. Several factors play into the onset of obesity, including economic, cultural, political and individual factors [10]. One theory suggests that at the individual level, obesity has been associated with lifestyle and eating habits and also various neuroendocrine disorders, like Cushing’s syndrome or thyroid disease [11]. Part of the lifestyle causes of obesity is the modern diet and decreased daily physical activity, which lead to a net positive intake of calories and therefore to increase in weight. The increased intake of high-energy and high-fat foods is attributed to changes in eating patterns and popularization of processed food. The decrease in physical activity is attributed to the sedentary nature of modern work and modes of transportation [12]. Also, energy balance theory – a widely accepted major component of the obesity equation – suggests that genetic and hormonal factors influence the predisposition of individuals to become overweight or obese. Following this theory, increasing amounts of available, cheap, unhealthful food exacerbate obesity rate in countries [13].

Obesity is a risk factor for the development of several diseases. Individuals who are overweight or obese are at a higher risk for a range of non-communicable diseases, including cardiovascular diseases, diabetes, and cancer, than those who are not overweight and obese [2]. In addition, overweight and obese individuals are at a higher risk of dying from those non-communicable diseases. For example, a study found that Asians who are overweight and obese have a higher cardiovascular mortality rate than those who are not overweight and obese [14]. Similarly, a recent study finds that maternal obesity is a risk factor for neonatal deaths [15]. Being overweight also increases risk during surgery. Increased BMI is associated with increased risk of surgical site infections following surgery, respiratory tract infection and venous thromboembolisms.

Overweight and obesity is not only an issue for high-income countries but also a tremendous concern in many low-income and middle-income countries. These countries are facing a “double burden” of disease [16]. For example, individuals from many East African countries are at risk for malaria and other infectious diseases at a young age. Then, they face the risks of cardiovascular diseases and diabetes due to high-fat and processed food diets [17] later in life.

### Positive and negative health externalities of economic flows

Factors leading to obesity are multifaceted, and this study suggests specific trade flows can be one factor contributing to obesity in countries. Current studies outline numerous relationships between trade, economic growth, food consumption, and health outcomes [18, 19]. Collectively, these studies suggest that economic flows can generate both positive and negative externalities with respect to health. An increase in trade – with the assumption that it leads to growth – generates positive externalities [1] and provides individuals with the resources to spend on health [20]. Furthermore, when the increase in trade leads to growth, governments can use the additional resources to improve sanitary conditions, increase education, and improve health outcomes related to communicable diseases. Indeed, Owen and Lu find that trade openness is associated with lower infant mortality rates and higher life expectancy [21]. Similarly, Bergh and Nilsson find that economic globalization increases life expectancy [22].

Concomitantly, trade can generate negative externalities for health. Increased trade flows are associated with an increase in the prevalence of non-communicable diseases and chronic illness. The literature highlights three linkages between trade and non-communicable diseases: 1) trade liberalization, 2) growth of transnational food corporations, and 3) global food advertising and promotion [5]. Trade liberalization refers to the elimination of quotas and reduction of tariffs. The growth of transnational food corporation linkage refers to the launch of a supermarket revolution and the rapid spread of fast food chains worldwide; this element intertwines with widespread change and influence over the global food supply. The global food advertising and promotion linkage highlights the aggressive advertising and the changing culture of food consumption.

Together, these three thee linkages increase the availability of unhealthful food and global diffusion of unhealthy lifestyles and health damaging products [5]. Following Popkin (2001) [23], Hawkes (2006) [4] refers to these processes as “nutrition transitions,” which stem from shifts in availability and affordability of certain foods. Thow suggests that trade liberalization influences the food consumption pattern through facilitating trade in specific goods and services and decreasing protection for domestic industry [7]. A qualitative study analyzes the relationship between trade policies and import of different categories of food and finds that changes in trade policies facilitated growing availability and consumption of meat, dairy products, processed foods, and imported fruits in Central American countries [7].

Similar to flow of goods, foreign direct investment (FDI) may impact health.^1^ Economies of scale and long shelf-life make processed food an attractive sector for FDI [24]. Furthermore, developed nations can take advantage of branding and marketing of popular Western culture through investing in processed food enterprises in developing countries. FDI in food processing and retailing increase the availability and affordability of specific foods [25]. For example, the availability of snack food in Central American countries mostly originates from FDI from the United States [7]. The literature finds that the removal of restrictions on FDI increases sales of sugary products. Using a natural experimental design, Schram et al. demonstrate that compared with the Philippines, Vietnam’s removal of restrictions on FDI increased sales of sugar-sweetened carbonated beverages. Difference-in-difference models that test pre/post differences in total sugar-sweetened carbonated beverages sales suggest that growth rate of sugar-sweetened carbonated beverages sales increased from 3.3% to 12.1% per capita per year [26].

Trade liberalization, and subsequent increase in trade flow and FDI, can generate resources for governments and individuals to improve health outcomes. However, trade liberalization also can be associated with negative externalities, such as an increase in availability of unhealthful foods, which then leads to rising obesity. While trade liberalization is to blame for the availability and affordability of these foods, it is not the whole story, as individuals also need to prefer these foods. As such, it is important to look at other factors that stimulate the consumption of unhealthful foods.

### Globalization, sugar and processed food imports, and obesity

While trade, economic development, and prosperity provide machinery and transportation tools that reduce calories use, various aspects of globalization arguably heighten the increase in calories intake, consumption of unhealthful food, and eventually increase the prevalence of overweight and obesity (e.g., Goryakin et al. [3]). We suggest that one of the channels through which globalization heightens the consumption of unhealthy food calories is increasing sugar and processed food imports. The increased imports of these food products is due to the simultaneous, interactive effect of three different components of globalization – economic, political, and social globalization [27]. These food, which often deviate from and disrupt the tradiatioanl diet, exacerbate the prevalence of overweight and obesity in countries.

Goryakin et al. [3] contribute to the literature by taking into account Dreher’s [27] categorization of globalization and highlighting that different types of globalization have varying effects on overweight and obesity in countries. Following qualitative evidence on the relationship between globalization and overweight and obesity (e.g. [4, 28, 29]) Goryakin et al. conduct econometric analyses on how different sub-components of globalization affect overweight in 887,000 women living in 56 developing countries between 1991 and 2009. These sub-components include: (1) the economic dimension – which includes long distance flows of goods, capital and services as well as information and perceptions that accompany market exchanges, (2) the political dimension that characterizes the diffusion of government policies internationally, and (3) the social dimension – which captures the spread of ideas, information, images, and people [27]. Goryakin et al. find that globalization, as a whole, is substantially and significantly associated with an increase in the individual propensity to be overweight among women. Moreover, the political and social components of globalization dominate the influence of the economic dimension [3].

One of the ways the interaction of these components of globalization manifests is through imports of sugar and processed food. Components of globalization are known to interact because political globalization forms alliances and political ties, which encourages further economic liberalization and economic globalization [30]; these increased interactions then catalyze social globalization and incentivize individuals to prefer products from specific countries and cultures. Imported food products stand out in the context of globalization for various reasons. First, imported food products are unique because it is one of the first things individuals purchase with available resources and funds. Second, food is generally more affordable than other products, such as a mobile phone, car, or brand name purses. Therefore, when individuals are socialized into purchasing products from Western countries, they are likely to purchase snacks and food first and foremost. Third, food is consumed by people of all age, sex, and race, and therefore is used at a higher volume per capita than most of the other products. As an example, while globalization and trade liberalization facilitated rising availability and consumption of processed food in Central American countries, developed nations simultaneously take advantage of social globalization and branding and marketing of popular Western culture to increase sales in processed food [7]. Interactively, these sub-components of globalization drive the rising imports of sugar and processed food products.

As such, imported food products should capture the interaction of the sub-components of globalization. More specifically, imports of sugar and processed food capture the unhealthful effect of imported food products. Therefore, globalization’s effect on overweight and obesity should manifest in the increasing flows of imported sugar and processed food.

The growth of processed food and sugar products is fastest in low and middle-income countries (LMIC). The growth is due to a swift nutrition transition from traditional diets – typically contain low sodium, saturated fat, and glycaemic indexes [4, 31] – to processed food. The reason individuals are choosing to consume these unhealthful food is because of the increasing availability and affordability of these food products. As a consequence, the presence of processed food, which are typically high in salt, fat, and sugar, lead to a high prevalence of overweight and obese individual in countries.

Looking specifically at soft drink consumption and overweight in 75 countries, Basu et al. [32] find that globally soft drinks consumption grew from 9.5 gal per person per year in 1997 to 11.4 gal in 2010. Additionally, an 1 % increase in soft drink consumption is associated with an additional 4.8 overweight adults per 100 adults. This finding is statistically significant in LMIC.

The effect of sugar and processed food on BMI is not news. Governments have been actively trying to curb consumption of these products [33]. However, it is crucial to recognize that it is not just economic globalization or trade liberalization that contributes to the rising imports of these products. Other aspects of globalization play a significant role.

In fact, political globalization and social globalization are associated with increased availability of sugar and processed food as well as obesity. Table 1 presents the correlation matrix of sugar imports, processed food imports, and dimensions of globalization. One can see that sugar imports are most highly correlated with political globalization (r = .44) then social globalization (r = .38) and is the least correlated with economic globalization (r = .21). On the other hand, processed food imports are most highly correlated with social globalization (r = .47) then political globalization (r = .45) and are the least correlated with economic globalization (r = .30).

**Table 1:** Correlation Matrix of Imports and Globalization

	Sugar Imports	Processed Food Imports	KOF	Economic Globalization	Political Globalization	Social Globalization
Sugar Imports	1.00					
Processed Food Imports	0.87	1.00				
KOF	0.42	0.50	1.00			
Economic Globalization	0.21	0.30	0.87	1.00		
Political Globalization	0.44	0.45	0.71	0.29	1.00	
Social Globalization	0.38	0.47	0.91	0.80	0.43	1.00

The pattern above further underlines the importance of distinguishing between different sub-components of globalization when evaluating health outcomes – as Goryakin et al. [3] show. More importantly, the pattern demonstrates that it is crucial to recognize that in the areas where these sub-components overlap, one is likely to see a significant increase in the effect of globalization on calorie consumption. Sugar and processed food imports are one of the areas that experience the impact of such overlap.

### Hypotheses on sugar and processed food imports and overweight and obesity

Qualitatively, the literature highlights various causal mechanisms linking economic flows to overweight and obesity rates. Existing quantitative studies test the effect of globalization and soft drink consumptions on the prevalence of overweight and obesity. However, to our knowledge, none of the studies present a quantitative test connecting import of processed food to the prevalence of overweight and obesity.

We concur with existing studies on the effect of globalization on obesity and extend these studies to examine if sugar and processed food imports are partially responsible for the effect of globalization on overweight and obesity. Given the literature and our theoretical framework, the observable implication is that an increase in sugar and processed food imports to a country should be associated with an increased prevalence of overweight and obesity in the country.

#### Hypothesis

An increase in sugar and processed food imports is associated with an increase in the prevalence of overweight and obesity in countries.

## Methods

To evaluate the hypothesis, we include 172 countries in our analyses. Out of 172 countries (Additional file 1: Appendix A), 95 countries are overweight or obese – that is the average BMI in these countries are equal to or greater than 25 kg/m^2^ for more than one year during the time frame of our dataset (Additional file 1: Appendix B). The number of the countries included (see Additional file 1: Appendix A) is limited by the available body mass index data and KOF globalization index data [27]. The time frame of the study is from 1995 to 2010. The descriptive statistics are in Table 2.

**Table 2:** Descriptive Statistics

Variable	Observations	Mean	Standard Deviation	Minimum	Maximum
BMI Average	2526	24.81397	2.436399	19.55	34.6
Sugar + Processed Food Import (log)	2526	13.0963	8.446789	0	22.8017
KOF	2526	52.56307	17.8071	16.27	92.37
GDP capita (log)	2526	7.684392	1.592659	3.998296	11.32885
ODA Aid (log)	2526	15.02257	7.932	0	23.93647
Health Expenditure per capita	2526	713.2088	1036.304	1.360238	8361.732
Population (log)	2526	15.47784	2.07135	9.755857	21.01431
Age Dependency	2526	64.55606	18.42898	16.98816	115.8486
Total Import (log)	2526	16.18403	10.45045	0	28.40051
Total Export (log)	2526	15.9242	10.31374	0	28.08703
Democracy	2526	.5796158	.493701	0	1
Interstate Conflict Severity	2526	24.47775	54.43787	0	480
Intrastate Conflict	2526	.1221101	.3274662	0	1
Countries: 172					
Year: 1995–2010					

### Dependent variables

The dependent variable in this study is *average body mass index (BMI)*. *BMI* is an index of weight-for-height. One can calculate the *BMI* for an individual by dividing the individual’s weight in kilograms by the square of the individual’s height in meters (kg/m2). The dataset is from the WHO Database. The WHO defines a person with BMI that is greater than or equal to 25 as overweight and a person with BMI that is greater than or equal to 30 as obese.^2^

### Independent variables

#### Import of sugar and processed food products

The sugar and processed food import variable is the dollar amount of imported sugar and processed food. We identified the food products that are considered as sugar and processed food using the Standard International Trade Classification (SITC) Revision 4. The sugar imports category (SITC number 06) includes: sugar, molasses, honey (SITC 061), and sugar confectionary (SITC 062). The processed food imports category includes Cereal preparation (SITC number 04), more specifically cereal preparations and preparations of flour or starch of fruits or vegetables (SITC 048), and Miscellaneous edible products and preparation (SITC number 09), which includes: margarine, shortening (SITC 091), and edible products and preparations (SITC 092). The information is from the United Nations Comtrade Database.^3^

### Control variables

We include the following variables to control for potential threats to validity: (1) KOF globalization index (2) GDP per capita, (3) economic aid, (4) health expenditure per capita, (5) population, (6) age dependency, (7) regime type, (8) interstate conflict, (9) intrastate conflict, (10) total import, and (11) total export. Similar to the independent variables, these control variables are lagged by one year to establish temporal precedence and show that the hypothesized cause occurs before the observed effect.

Following Goryakin et al. [3], total globalization is measured using the Konjunkturforschungsstelle (KOF) Index of Globalization [27], which is an indicator developed by the Swiss Economic Institute. The indicator aggregates the economic, political, and social dimensions of globalization.^4^ The data is from Dreher (2006) [27], but updated in 2015.

GDP per capita captures the availability of resources to purchase goods, such as food and imported products. Furthermore, Lieberman (2007) [34] suggests that GDP^5^ per capita influences a state’s ability and willingness to spend resources on health prevention. Controlling for GDP per capita ensures that there is not varying rates of regression to the mean of the different nations simply because of varying social situations.

Foreign economic aid is included to control for the fact that some states might be more or less capable because of the presence or absence of significant outside assistance [35]. Foreign aid can subsidize a less capable state and allow the government to carry out its duties when they normally cannot. The data for foreign aid are from the World Bank 2015 dataset.

It is logical to expect that a larger population would consume a larger amount of sugar and processed food imports than a smaller population. The inclusion of population size in our models allows us to control the rate of consumption. We also include healthcare expenditure per capita as a control variable as studies show healthcare expenditure affects health outcomes [36]. The data are from the World Bank Dataset.

The effect of trade on health outcome could be due to economic liberalization and the improvement in the standard of living. In this case, the total volume of imports and exports should significantly affect health outcome. Therefore, we control for *total imports* and *total exports* to ensure that it is the import of processed food that is generating higher overweight and obesity weight.

Age is a risk factor for many health outcomes [37]. The age distribution in a country affects the disease distribution, mortality rate, and productivity of the population. Therefore, we control for the varying age structure in countries using the percentage of dependents (the share of individuals below the age of 16 and above the age of 65) to measure age structure.

Democracies on average are associated with higher level of health services than autocracies [38]. Controlling for regime type monitors the effect of different governmental processes [34]. We employ Cheibub, Gandhi, and Vreeland’s (2010) [39] dataset on regimes to control for regime type.^6^

Conflicts can reduce the availability of food for consumption. To account for the effect of conflicts, we employ *militarized interstate disputes (MID)* to capture the effect of the guns-versus-butter dilemma, which hypothesizes the tradeoff between military and social spending [40, 41], plus the direct impact of violence.^7^ We also include a binary variable for intrastate conflict, where 0 indicates an absence of intrastate conflict and 1 indicates the presence of an intrastate conflict in a given country year.

### Estimation strategy

Following Goryakin et al. [3], we employ both Ordinary Least Squares (OLS) models and country-specific fixed effects (FE) models to examine the relationship between sugar and processed foods imports, globalization, and average BMI. Given that our study aims to highlight the variation across countries, we focus on the FE models, which provide additional control for unobservable country-specific influences [42], and we provide the results from the OLS models in the appendices. The FE models include country dummy variables as extra regressors, allowing variation in these effects while not imposing the strict condition that regressors are uncorrelated with fixed effects. The characteristics of FE models are especially important in our study because there may be country-specific food consumption patterns and food production patterns that are not captured by the control variables; FE models address the issue. We also use robust standard errors clustered by country, which allows for the correction for serial correlations and panel heteroskedasticity. In the analyses, the coefficient sizes are generally smaller in the FE models, but the explanatory power of these models, as indicated by larger R^2^, are greater than in the OLS models.

## Results and discussion

We first evaluate the hypothesis on the relationship between sugar processed food imports and average BMI of all countries in our sample. The FE models in Table 3 take into account *BMI* in all 172 countries in our dataset (OLS comparison models are in Additional file 1: Appendix C), whether they are overweight or obese or not. Model 1 in Table 3 examines the effect of KOF on average BMI. The model shows that a one-index point increase in KOF is associated with a 0.013 increase in average BMI. In Model 2, we evaluate if import and exports are more refined explanatory variables for average BMI. The results from Model 2 indicate that after controlling for general imports and exports, which both generated statistically significant coefficients, the coefficient for KOF diminishes. This finding indicates that general imports volume and exports volume capture part of the effect of globalization, which is no surprise, as KOF specifically measures the economic dimension of globalization. In Model 3, we examine if sugar and processed food imports is associated with average BMI. We find that a one-unit increase in sugar and processed food imports (log) is associated with a 0.004 increase in average BMI. Translating the log-transformed variable into substantively meaningful results: a 10% increase in average import is associated with approximately 0.0002 increase in average BMI and a 50% increase in average import is associated with 0.0007 increase in average BMI.

**Table 3:** Average BMI Worldwide

	(1) FE	(2) FE	(3) FE	(4) FE
VARIABLES	BMI Average	BMI Average	BMI Average	BMI Average
Sugar and Processed Food Import (log)			0.004***	0.001
			(0.001)	(0.010)
KOF	0.013***	0.011***	0.010***	0.011***
	(0.002)	(0.002)	(0.001)	(0.002)
GDP capita (log)	0.652***	0.645***	0.567***	0.645***
	(0.039)	(0.038)	(0.034)	(0.038)
ODA Aid (log)	0.002	0.002	0.002*	0.002
	(0.001)	(0.001)	(0.001)	(0.001)
Health Expenditure per capita	0.000***	0.000***	0.000***	0.000***
	(0.000)	(0.000)	(0.000)	(0.000)
Population (log)	2.129***	2.154***	2.103***	2.154***
	(0.072)	(0.071)	(0.066)	(0.071)
Age Dependency	−0.015***	−0.015***	−0.016***	− 0.015***
	(0.001)	(0.001)	(0.001)	(0.001)
Total Import (log)		0.014***		0.014*
		(0.002)		(0.008)
Total Export (log)		−0.009***		−0.009***
		(0.002)		(0.002)
Democracy	−0.084***	−0.087***	− 0.057**	−0.087***
	(0.028)	(0.027)	(0.023)	(0.027)
Interstate Conflict Severity	−0.000	− 0.000	−0.000	− 0.000
	(0.000)	(0.000)	(0.000)	(0.000)
Intrastate Conflict	−0.062**	−0.068***	0.013	−0.068***
	(0.026)	(0.025)	(0.010)	(0.025)
Constant	−13.145***	−13.491***	−13.011***	−13.488***
	(1.211)	(1.196)	(1.140)	(1.197)
Observations	2408	2408	2109	2109
R-squared	0.746	0.753	0.795	0.753
Number of Countries	172	172	172	172

Standard errors in parentheses

***p < 0.01, **p < 0.05, *p < 0.1

In addition to our variables of focus, we also find that democracies are associated with a lower average BMI than non-democracies. Interstate conflict severity has a positive relationship with average BMI, although the coefficients are not statistically significant. Intrastate conflicts, as expected, have a negative relationship with BMI. All other coefficients are as expected.

We recognize that the effect of sugar and processed food imports cannot be evaluated independently from general imports and exports. Thus, in Model 4, we include all of the trade variables: sugar and processed food imports, general imports, and general exports. The results from this model suggest that an increase in *total import* is associated with an increase in average *BMI*. On the other hand, *total export* is associated with a reduction in average BMI. Once we take into account the general pattern of trade, the import of sugar and processed food no longer has a statistically significant effect on average BMI in this worldwide sample. The results are not surprising; Goryakin et al. [3] outline that changes in BMI has different implications. An increase from a BMI of 18.5 to a BMI of 19 (i.e. from malnourishment to normal is different from a BMI of 24 to 25 (i.e. normal to overweight).

Given the different implications of changing BMI, we turn our attention to the shift in average BMI in overweight and obese countries. Existing studies utilize two main approaches when analyzing overweight and obesity as a dependent variable. For example, Goryakin et al. [3] generate a binary variable for overweight, when individuals have BMI of over 25 kg/m^2^. Alternatively, De Vogli et al. (2013) [43] use a continuous BMI variable. The binary variable resolves the issue of varying implications of increasing BMI and avoids convoluting the analysis on the relationship between imports and average BMI. However, collapsing a continuous variable into a binary variable eliminates information on the nuances and changes in average BMI.

To balance the advantages and disadvantages of the two approaches, we include additional analyses that focus on a subsample of all overweight and obese countries. The results of the analyses on this subsample inform us the relationship between imports and rising average BMI in countries that are already facing the issue of overweight and obesity. The analysis thus highlights how imports of sugar and processed food may exacerbate the prevalence of overweight and obesity.

In Table 4, we evaluate the effect of sugar and processed food imports on average BMI in overweight and obese countries. Model 1 in Table 4 provides the baseline fixed-effects model, which does not include *sugar and processed food import* variable. We find – congruent with results from Goryakin et al. [3] – the KOF variable is statistically significant. Model 2 in Table 4 is a FE model that includes *sugar and processed food import* variable and evaluates its relationship with average BMI (OLS comparison models are in Additional file 1: Appendix D). One-unit increase in *sugar and processed food import (log)* is associated with a .085 increase in average BMI. Translating the log-transformed variable into substantively meaningful results: 10% increase in import is associated with approximately 0.004 increase in average BMI in the sample of overweight and obese countries. The effect of sugar and processed food imports on average BMI is substantial.

**Table 4:** Average BMI in Overweight and Obese Countries

	(1) FE	(2) FE
	BMI Average	BMI Average
Variables		
Sugar and Processed Food Import (log)		0.085***
		(0.027)
KOF	0.009***	0.008***
	(0.002)	(0.002)
GDP capita (log)	0.911***	0.917***
	(0.070)	(0.070)
ODA Aid (log)	0.004**	0.004**
	(0.002)	(0.002)
Health Expenditure per capita	0.000***	0.000***
	(0.000)	(0.000)
Population (log)	2.538***	2.533***
	(0.126)	(0.125)
Age Dependency	−0.021***	−0.022***
	(0.003)	(0.003)
Total Import (log)	0.018***	−0.050**
	(0.003)	(0.022)
Total Export (log)	−0.013***	−0.014***
	(0.003)	(0.003)
Democracy	−0.192***	−0.213***
	(0.074)	(0.074)
Interstate Conflict Severity	0.000	0.000
	(0.000)	(0.000)
Intrastate Conflict	−0.060	−0.073
	(0.052)	(0.052)
Constant	−19.373***	−19.286***
	(2.273)	(2.265)
Observations	1423	1423
R-squared	0.736	0.738
Number of Countries	112	112

Standard errors in parentheses

***p < 0.01, **p < 0.05, *p < 0.1

KOF still has a statistically significant relationship with average BMI; however, the coefficient for KOF decreased by 0.001 when we include our variable of interest *sugar and processed food imports* in the model (Table 4 Model 2). The reduction in coefficient size for KOF indicates that our explanatory variable partially explains why KOF is associated with increased average BMI. In other words, part of why globalization is associated with increased average BMI is due to the increase in imports of unhealthful food.

With the exception of regime type and conflicts, coefficients for the other control variables are as expected. Democracies are associated with a lower average BMI than autocracies. And, in the sample of overweight and obese countries both interstate and intrastate conflicts have no statically significant effect on average BMI.

In summary, the models demonstrate that sugar and processed food imports are part of the reason why globalization and trade liberalization increase the prevalence of overweight and obesity in countries. We evaluate the effect of sugar and processed food imports while controlling for globalization index and general imports and exports; these variables remain significant, albeit with a smaller effect because of the inclusion of our main variable of interest. Our results thus add to previous studies and highlight that sugar and processed food imports are crucial factors influencing the prevalence of overweight and obesity worldwide.

### Fiji

We utilize the case of Fiji to illustrate our statistical findings on the global pattern between sugar and processed food imports and average BMI. Fiji is an informative case as it is one of the many islands in the Melanesian Pacific region which face a worsening epidemic of overweight and obesity as they experience a continuous cycle of food dependence due to globalization [44]. The situation in Fiji demonstrates the severe effect of globalization, trade liberalization, imported products, on the prevalence of overweight and obesity that are occurring in some countries.

Fiji has a very active trade agreement profile since the 1980s. Various multilateral, bilateral, regional, and global trade agreements contribute to trade liberalization efforts (see Additional file 1: Appendix F for the list of agreements), which reduce tariff barriers of food commodities, among other products. As a result, Fiji, with lower tariffs and an import-oriented economy, imports a higher number of processed food and sugary drink imports as compared to countries with high tariff and protection.

Since trade liberalization, the increase of imports in breakfast cereals, confectionary, and pastry items nearly quadrupled in 15 years [45]. Furthermore, Fiji maintains trade liberalization policies and keep sugar-based beverages (SBBs) duty-free for most favored nationals in the World Trade Organization (WTO). The action is associated with an increase in SBBs in Fiji [46].

The increase in food imports is linked to the rising consumption of processed and packaged food in Fiji [47, 48]. Fiji’s overweight and obese populations are apparent. In addition, there is a rise in obesity-related Type 2 Diabetes Mellitus (T2DM). Future trends in obesity and T2DM prevalence are expected to increase in Fiji and have raised concerns for morbidity and mortality related complications [49]. These statistics are attributed to a shift in traditional diets and a nutritional transition in the Fijian food environment [50].

Concern over the prevalence of obesity and rise of non-communicable diseases have prompted policymakers to address these outcomes through trade-related policies.^8^ Increasing duties and import taxes may act as a trigger mechanism to control the volume of sugary and processed food; yet, in the case of Fiji, policy implementation and limited impact assessment have not contributed to a decrease in imports or reduction in obesity. The ineffectiveness of these policies may be due to the social component of globalization, which normalized the frequent consumption of these foods. A higher tariff or broader education campaigns may be necessary to alter individual preference and incentivize individuals to reduce their consumptions of sugar and processed food.

### Synthetic control method

To further highlight the relationship between sugar and processed food imports and average BMI, we employ a synthetic control method [51, 52] to calculate a counterfactual average BMI in Fiji, which assumes that Fiji did not participate in a series of trade liberalization and experienced an increase in sugar and processed food imports. Using a synthetic control method (SCM) framework,^9^ we generate synthetic control units, which are counterfactuals [52]. The counterfactual allows for the inference of what would be the average BMI in Fiji without the high inflow of sugar and processed food. The control unit in the SCM is constructed using a weighted average of all potential comparison units, which is from a donor pool of countries. We analyze the average BMI of Fiji compared to an estimated control group because a combination of comparison units tend to match more accurately the characteristics of the unit (in our case Fiji) than any single comparison unit alone [53].

The unit of analysis is country-year. To build the counterfactual control unit, we use the inflow of sugar and processed food (log transformed) in countries that have an inflow of less than one standard deviation (8.45) from the sample mean (13.10). We restricted the pool of control unit countries to countries in the same region as Fiji.^10^

The goal is to match the Fiji’s average BMI (over time) to the average BMI (over time) for the synthetic control, the weighted average of countries in the donor pool; that is, to compose average BMIs that are relatively equal to that of Fiji’s average BMI. Each country in the donor pool gets a weight that reflects its similarity to Fiji. We include the following covariates: KOF, GDP capita (log), ODA aid (log), health expenditure per capita, population (log), age dependency, general import (log), general export (log), democracy, interstate conflict severity, and intrastate conflict. The result is a synthetic control unit with average BMI patterns that closely resemble Fiji’s actual average BMI in the years leading up to a series of trade liberalizations (starting around 2000), resulting in an increase in sugar and processed food imports. The average BMI of Fiji and the synthetic control average BMI may then diverge in the years after increased in imports.

**Fig. 1:**
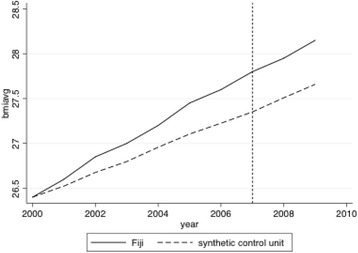
Trends in BMI: Fiji versus synthetic Fiji

Figure 1 supports the qualitative description and the current literature on the cause of increased prevalence of overweight and obesity in Fiji; the increased prevalence is associated with trade agreements and increased imports of sugar and processed food. In this figure, the dashed line is the synthetic control unit calculated using the sample of countries similar to Fiji. The solid line is the actual trend of average BMI in Fiji from 2000 to 2009. The difference between the two lines illustrates the impact of sugar and processed food import on average BMI in Fiji. The dotted vertical line indicates the year 2007, which one can see the actual average BMI in Fiji that year is approximately 27.75. On the other hand, if Fiji did not experience a surge of imported sugar and processed food, the average BMI would be approximately 27.25. The counterfactual thus suggest that sugar and processed food imports are associated with a .5 increase in average BMI in Fiji.

## Conclusion

Existing literature provides abundant evidence that globalization is associated with increased prevalence of overweight and obesity; however, specific mechanisms connecting globalization and prevalence of overweight and obesity have not been examined quantitatively. Following the linkages outlined by previous studies, we examine if the import level of sugar and processed food products is one of the mechanisms through which globalization increases overweight and obesity rates in countries worldwide. We find that sugar and processed food imports have a statistically significant relationship with increasing average BMI in countries that are overweight or obese.

Examining countries worldwide – including countries that have consistently high prevalence of overweight and obesity and countries that do not – we find that the level of sugar and processed food imports is associated with a rise in average BMI. Nevertheless, once we take into account the general pattern of trade, the import of sugar and processed food no longer has a statistically significant relationship with average BMI. This pattern is not surprising as trade liberalization can increase wealth and improve the living standard of previously malnourish countries, leading to these countries obtaining an average BMI that is considered healthful.

Therefore, we specifically evaluate the effect of sugar and processed food imports on average BMI in overweight and obese countries. The model shows that a unit increase in *sugar and processed food import (log)* is associated with .085 increase in average BMI. Translating the log-transformed variable into substantively meaningful results, we find that a 10% increase in imports of sugar and processed food is associated with approximately 0.004 increase in average BMI.

In addition to the statistically and substantively significant association between sugar and processed food imports and average BMI, we qualitatively evaluate the case of Fiji, which highlights how imports of processed foods can impact obesity in a specific country. The involvement of Fiji in a variety of trade agreements created a market with few restrictions on the importation of processed and sugary food, which ultimately contributed to a dietary transition [4]. The availability of sugar and processed food and the dietary transition exacerbated the prevalence of overweight and obesity in Fiji.

We further evaluate the link between increased sugar and processed food imports and a rise in average BMI in Fiji through a synthetic control method. The pattern from the synthetic control analysis shows the comparison between Fiji’s current average BMI and a counterfactual average BMI, assuming that Fiji did not experience the influx of sugar and processed food. The counterfactual average is significantly lower than that of the realistic growth in average BMI in Fiji. Our findings are consistent with literature that suggests a strong linkage between sugar and processed food imports and obesity in Fiji.

This study adds to the existing literature that suggests globalization is a driver of dietary transitions and rise in BMI by demonstrating that sugar and processed food imports are important mechanisms through which globalization and trade liberalization influence the prevalence of overweight and obesity. We first show a global pattern of sugar and processed food imports and average BMI. We then focus on countries that are consistently overweight and obese and highlight that these countries are more negatively impacted by sugar and processed food imports than countries with lower prevalence of overweight and obesity. The reason for the varying pattern is that in the sample, which includes all countries with available data, contains developing countries that are experiencing increasing wealth. These countries are moving from average BMIs that are considered malnourished (less than 18.5 kg/m^2^) to normal, healthful BMIs (18.5 to 24.9 kg/m^2^). In the sample of overweight and obese countries, the unhealthful BMI is exacerbated by the inflow of imports of sugar and processed food. This pattern of exacerbated level of overweight and obesity is highlighted by the case of Fiji.

While our study underlines the relationship between sugar and processed food imports, our study inevitably suffers from several limitations. First, due to data availability, we were unable to include all countries worldwide. Second, there may be country-specific factors that were not included in our model; nevertheless, the fixed-effect approach should take into account country-specific factors not captured by the control variables. Third, obesity is multifaceted; as such, there are many mechanisms that are associated with the prevalence of obesity in countries. This study is only scratching the surface of understanding the relationship between global factors and obesity; further analyses need to be conducted to better understand the connection.

It is estimated that $150 billion US dollars or £100 billion / €118 billion [13] per year of health care budget are spent on obesity associated complications and pathologies. Obesity is a multifactorial disease but it is widely accepted that lifestyle and the modern diet play an important part in its aetiology; these lifestyle and diet preferences are shaped partly by societal values and the direct and indirect influence of globalization. Unfortunately, many diets and excise plans as well as many economic policies have not proven to be effective at reducing consumption of unhealthful food. The widespread prevalence of overweight and obesity globally requires attention from different international arenas. The absence of concurrent policies may be one of the reasons why many diet regiments, exercise plans, and economic policies independently have not been effective. We reason that due to the interconnection between different components of globalization and overweight and obesity there is a need for increasing collaboration between international organizations such as the WHO and the WTO. A one-sided approach may not be sufficient in ameliorating a situation where, at the very least, requires modification in trade policies and health policies.

Concomitantly, the intricate incentive for purchasing sugar and processed food imports suggests that it may be fruitful for research and policy to focus on combating obesity from a local, city, or grassroots level. In the absence of sufficient global policy on overweight and obesity, local, city, or grassroots movements may allow for more individualized policies, which are more effective at targeting individual incentives for purchasing and consuming sugar and processed food than any one-sided global action. Implementation of well-informed public health measures by governments to guard their populations against obesogenic environments is necessary.

## Additional files

Additional file 1: **Appendix A**. Countries included in the Analysis. **Appendix B**. Overweight and Obese Countries (more than one year during the time frame) Included in the Analysis. **Appendix C**. Comparing OLS Models with FE Models. **Appendix D**. Comparing OLS Models with FE Models for Overweight and Obese Countries. **Appendix E**. Models examining Sub-components of Globalization. **Appendix F**. Fiji Trade Agreement Profile. (DOC 186 kb)

## Abbreviations

BMI: Body mass index
FDI: Foreign direct investment
FE: Fixed effects
GDP: Gross domestic production
KOF: Konjunkturforschungsstelle Index of Globalization
LMIC: Low and middle-income countries
MID: Militarized interstate dispute
OLS: Ordinary Least Squares
SBBs: Sugary-based berverages
SCM: Synthetic control method
SITC: Standard International Trade Classification
T2DM: Type 2 Diabetes Mellitus
WHO: World Health Organization
WTO: World Trade Organization

## Footnotes

1 Similar to trade flow, FDI generates both positive externalities and negative externalities. With the increasing capital in countries, governments should be able to improve the sanitary conditions, reduce communicable diseases, and educate the public about health. The increase in general FDI should reduce communicable diseases while an increase in FDI focused on the food sector should increase the prevalence of chronic illnesses.

2
http://www.who.int/mediacentre/factsheets/fs311/en/

3
http://comtrade.un.org/data/

4 The economic globalization index is a composite of the following variables: trade (in percent of GDP), foreign direct investment stocks (in percent of GDP), portfolio investment (in percent of GDP), income payments to foreign nationals (in percent of GDP), hidden import barriers, mean tariff rate, taxes on international trade, and capital account restrictions. The political globalization index is a composite of the following: number of foreign embassies in a given country, membership in international organizations, participation in U.N. Security Council missions, number of signed international treaties. The social globalization index is based on the following variables: telephone traffic transfers, international tourism foreign population, international letters, internet users, TVs per 1000 people, trade in newspapers, number of McDonald’s restaurants, number of Ikea per capita, and trade in books (percent of GDP).

5 GDP also serves as a proxy for education level. Education plays a role in individual’s ability to make health-conscious decisions. The primary education level indicator is from the World Bank dataset. The data ranges from 1960 to 2010 but are only available every two to three years. To avoid listwise deletion, we employ GDP as a proxy.

6 In their dataset, “a regime is considered a democracy if the executive and the legislature is directly or indirectly elected by popular vote, multiple parties are allowed, there is de facto existence of multiple parties outside of regime front, there are multiple parties within the legislature, and there has been no consolidation of incumbent advantage.”

7 Given that we aim to measure the severity of conflict on health outcomes, we employ Maoz’s (1982) method of converting the MID categories into an interval severity scale [28]. The possible severity score for each military action ranges from 0 to 100. The total severity score for each country-year sums the severity score for all military action a country engages in a given year. As a result, the severity variable can be above 100.

8 Since 2000, Fiji has enacted laws ranging from banning the sale of mutton flaps to requiring high-fat product package labeling. However, despite these measures, the observed rise in BMI suggests minimal impact in the reduction of obesity. For example, in 2006, Fiji enacted a 5% import duty and 5 cents per liter local excise duty on sweetened beverages. This policy aimed to reduce the influx of imports and increase the cost of sugary beverages. Nevertheless, a higher cost was not associated with decreased consumption or a reduction in imports Similar increases on duties for palm oil, monosodium glutamate, and non-local produce were repeated in 2012 without desired outcomes [33].

9 SCM has several advantages. First, SCM is transparent as the weight and contribution of each comparison unit in the sample is made explicit. Second, SCM helps mitigate the problem of confounding factors. By choosing a qualitatively reasonable donor pool, we control for many potential confounding factors without having to use them as the basis for a match. In addition, we focused on countries in the same region as Fiji. This reduces the risk of omitted variable bias. Exogenous and unrelated changes are less likely to be driving estimates. We use the synth command in Stata to implement the methodology [52].

10 A sample of countries, which includes Papua New Guinea, Vanuatu, Solomon Islands, Nauru, Marshall Islands, and Micronesia, constitute the control group.
