# Supplementary material for: The effect of sugar and processed food imports on the prevalence of overweight and obesity in 172 countries
Source: Global Health. 2018 Apr 14;14:35. doi: 10.1186/s12992-018-0344-y (PMC5899384; doi:10.1186/s12992-018-0344-y)
Supplement: Supplementary file 1 — Appendix A. Countries included in the Analysis. Appendix B. Overweight and Obese Countries (more than one year during the time frame) Included in the Analysis. Appendix C. Comparing OLS Models with FE Models. Appendix D. Comparing OLS Models with FE Models for Overweight and Obese Countries. Appendix E. Models examining Sub-components of Globalization. Appendix F. Fiji Trade Agreement Profile. (DOC 186 kb) [file 12992_2018_344_MOESM1_ESM.doc]

Appendix A: Countries included in the Analysis

| Afghanistan  Albania  Algeria  Angola  Antigua and Barbuda  Argentina  Armenia  Australia  Austria  Azerbaijan  Bahamas  Bahrain  Bangladesh  Barbados  Belarus  Belgium  Belize  Benin  Bhutan  Bolivia  Bosnia and Herzegovina  Botswana  Brazil  Brunei Darussalam  Bulgaria  Burkina Faso  Burundi  Cambodia  Cameroon  Canada  Cape Verde  Central African Republic  Chad  Chile  China  Colombia  Comoros  Congo, Dem. Rep.  Congo, Rep.  Costa Rica  Cote d'Ivoire  Croatia  Cuba  Cyprus  Czech Republic  Denmark  Djibouti  Dominica  Dominican Republic | Ecuador  Egypt, Arab Rep.  El Salvador  Equatorial Guinea  Eritrea  Estonia  Ethiopia  Fiji  Finland  France  Gabon  Gambia  Georgia  Germany  Ghana  Greece  Grenada  Guatemala  Guinea  Guinea-Bissau  Guyana  Haiti  Honduras  Hungary  Iceland  India  Indonesia  Iran, Islamic Rep.  Iraq  Ireland  Israel  Italy  Jamaica  Japan  Jordan  Kazakhstan  Kenya  Kiribati  Kuwait  Kyrgyz Republic  Lao PDR  Latvia  Lebanon  Lesotho  Liberia  Libya  Lithuania  Luxembourg | Macedonia, FYR  Madagascar  Malawi  Malaysia  Maldives  Mali  Malta  Mauritania  Mauritius  Mexico  Moldova  Mongolia  Montenegro  Morocco  Mozambique  Myanmar  Namibia  Nepal  Netherlands  New Zealand  Nicaragua  Niger  Nigeria  Norway  Oman  Pakistan  Palau  Panama  Papua New Guinea  Paraguay  Peru  Philippines  Poland  Portugal  Qatar  Romania  Russian Federation  Rwanda  Samoa | Sao Tome and Principe  Saudi Arabia  Senegal  Seychelles  Sierra Leone  Singapore  Slovak Republic  Slovenia  Solomon Islands  Somalia  South Africa  Spain  Sri Lanka  St. Kitts and Nevis  St. Lucia  St. Vincent and the Grenadines  Sudan  Suriname  Swaziland  Sweden  Switzerland  Syrian Arab Republic  Tajikistan  Tanzania  Thailand  Timor-Leste  Togo  Tonga  Trinidad and Tobago  Tunisia  Turkey  Turkmenistan  Uganda  Ukraine  United Arab Emirates  United Kingdom  United States  Uruguay  Uzbekistan  Vanuatu  Venezuela, RB  Vietnam  Yemen, Rep  Zambia  Zimbabwe |
| --- | --- | --- | --- |

Appendix B: Overweight and Obese Countries (more than one year during the time frame) Included in the Analysis

| Albania  Algeria  Argentina  Armenia  Australia  Austria  Azerbaijan  Bahamas, The  Bahrain  Barbados  Belarus  Belgium  Belize  Bolivia  Bosnia and Herzegovina  Brazil  Bulgaria  Canada  Cape Verde  Chile  Colombia  Costa Rica  Croatia  Cyprus  Czech Republic  Denmark  Dominican Republic | Ecuador  Egypt, Arab Rep.  El Salvador  Estonia  Fiji  Finland  France  Gabon  Germany  Greece  Guatemala  Guyana  Honduras  Hungary  Iceland  Iran, Islamic Rep.  Ireland  Israel  Italy  Ivory Coast  Jamaica  Jordan  Kazakhstan  Kuwait  Kyrgyz Republic | Latvia  Lebanon  Lithuania  Luxembourg  Macedonia, FYR  Malaysia  Malta  Mauritius  Mexico  Moldova  Mongolia  Montenegro  Morocco  Netherlands  New Zealand  Nicaragua  Norway  Oman  Panama  Papua New Guinea  Paraguay  Peru  Poland  Portugal  Qatar  Romania  Russian Federation | Saudi Arabia  Slovak Republic  Slovenia  South Africa  Spain  Suriname  Swaziland  Sweden  Switzerland  Syrian Arab Republic  Trinidad and Tobago  Tunisia  Turkey  Ukraine  United Arab Emirates  United States  Uruguay  Uzbekistan  Vanuatu  Venezuela  Yemen, Rep |
| --- | --- | --- | --- |

Appendix C: Comparing OLS Models with FE Models

|  | (1) OLS | (2) FE | (3) OLS | (4) FE | (5) OLS | (6) FE |
| --- | --- | --- | --- | --- | --- | --- |
| VARIABLES | BMI Average | BMI Average | BMI Average | BMI Average | BMI Average | BMI Average |
|  |  |  |  |  |  |  |
| Sugar and Processed Food Import (logged) |  |  |  |  | 0.017 | 0.001 |
|  |  |  |  |  | (0.023) | (0.010) |
| KOF | 0.022*** | 0.013*** | 0.021*** | 0.011*** | 0.021*** | 0.011*** |
|  | (0.004) | (0.002) | (0.004) | (0.002) | (0.004) | (0.002) |
| GDP capita (logged) | 0.866*** | 0.652*** | 0.853*** | 0.645*** | 0.855*** | 0.645*** |
|  | (0.048) | (0.039) | (0.049) | (0.038) | (0.049) | (0.038) |
| ODA Aid (logged) | 0.070*** | 0.002 | 0.069*** | 0.002 | 0.069*** | 0.002 |
|  | (0.007) | (0.001) | (0.007) | (0.001) | (0.007) | (0.001) |
| Health Expenditure per capita | -0.000*** | 0.000*** | -0.000*** | 0.000*** | -0.000*** | 0.000*** |
|  | (0.000) | (0.000) | (0.000) | (0.000) | (0.000) | (0.000) |
| Population (logged) | -0.339*** | 2.129*** | -0.351*** | 2.154*** | -0.352*** | 2.154*** |
|  | (0.021) | (0.072) | (0.022) | (0.071) | (0.022) | (0.071) |
| Age Dependency | -0.019*** | -0.015*** | -0.019*** | -0.015*** | -0.019*** | -0.015*** |
|  | (0.003) | (0.001) | (0.003) | (0.001) | (0.003) | (0.001) |
| Total Import (logged) |  |  | -0.005 | 0.014*** | -0.020 | 0.014* |
|  |  |  | (0.012) | (0.002) | (0.023) | (0.008) |
| Total Export (logged) |  |  | 0.013 | -0.009*** | 0.013 | -0.009*** |
|  |  |  | (0.012) | (0.002) | (0.013) | (0.002) |
| Democracy | 0.049 | -0.084*** | 0.034 | -0.087*** | 0.036 | -0.087*** |
|  | (0.069) | (0.028) | (0.070) | (0.027) | (0.070) | (0.027) |
| Interstate Conflict Severity | 0.004*** | -0.000 | 0.004*** | -0.000 | 0.004*** | -0.000 |
|  | (0.001) | (0.000) | (0.001) | (0.000) | (0.001) | (0.000) |
| Intrastate Conflict | -0.383*** | -0.062** | -0.372*** | -0.068*** | -0.368*** | -0.068*** |
|  | (0.102) | (0.026) | (0.103) | (0.025) | (0.103) | (0.025) |
| Constant | 22.518*** | -13.145*** | 22.778*** | -13.491*** | 22.778*** | -13.488*** |
|  | (0.623) | (1.211) | (0.640) | (1.196) | (0.640) | (1.197) |
|  |  |  |  |  |  |  |
| Observations | 2,408 | 2,408 | 2,109 | 2,109 | 2,109 | 2,109 |
| R-squared | 0.552 | 0.746 | 0.553 | 0.753 | 0.553 | 0.753 |
| Number of Countries | 172 | 172 | 172 | 172 | 172 | 172 |

Appendix D: Comparing OLS Models with FE Models for Overweight and Obese Countries

|  | (1) OLS | (2) FE | (3) OLS | (4) FE |
| --- | --- | --- | --- | --- |
|  | BMI Average | BMI Average | BMI Average | BMI Average |
| VARIABLES |  |  |  |  |
|  |  |  | 0.079*** | 0.085*** |
| Sugar and Processed Food Import (logged) |  |  | (0.025) | (0.027) |
|  | -0.009** | 0.009*** | 0.022*** | 0.008*** |
| KOF | (0.004) | (0.002) | (0.004) | (0.002) |
|  | 0.389*** | 0.911*** | 0.930*** | 0.917*** |
| GDP capita (logged) | (0.050) | (0.070) | (0.049) | (0.070) |
|  | -0.006 | 0.004** | 0.039*** | 0.004** |
| ODA Aid (logged) | (0.006) | (0.002) | (0.008) | (0.002) |
|  | 0.015** | 0.004** | 0.058*** | 0.004** |
| Health Expenditure per capita | (0.006) | (0.002) | (0.008) | (0.002) |
|  | 0.000 | 0.000*** | -0.000*** | 0.000*** |
| Population (logged) | (0.000) | (0.000) | (0.000) | (0.000) |
|  | -0.181*** | 2.538*** | -0.373*** | 2.533*** |
| Age Dependency | (0.021) | (0.126) | (0.022) | (0.125) |
|  | 0.021*** | -0.021*** | -0.019*** | -0.022*** |
| Total Import (logged) | (0.003) | (0.003) | (0.003) | (0.003) |
|  | 0.020* | 0.018*** | -0.072*** | -0.050** |
| Total Export (logged) | (0.011) | (0.003) | (0.023) | (0.022) |
|  | -0.021* | -0.013*** | 0.010 | -0.014*** |
| Democracy | (0.012) | (0.003) | (0.012) | (0.003) |
|  | -0.939*** | -0.192*** | 0.041 | -0.213*** |
| Interstate Conflict Severity | (0.070) | (0.074) | (0.069) | (0.074) |
|  | 0.002*** | 0.000 | 0.003*** | 0.000 |
| Intrastate Conflict | (0.001) | (0.000) | (0.001) | (0.000) |
|  | -0.475*** | -0.060 | -0.348*** | -0.073 |
| Constant | (0.129) | (0.052) | (0.102) | (0.052) |
|  | 25.846*** | -19.373*** | 22.210*** | -19.286*** |
|  | (0.585) | (2.273) | (0.637) | (2.265) |
| Observations |  |  |  |  |
| R-squared | 1,423 | 1,423 | 1,423 | 1,423 |
| Number of Countries | 0.282 | 0.736 | 0.563 | 0.738 |
| Number of ccode | 112 | 112 | 112 | 112 |

Appendix E: Models examining Sub-components of Globalization

|  | (5) | (2) | (3) | (4) | (1) |
| --- | --- | --- | --- | --- | --- |
| VARIABLES | BMI Average | BMI Average | BMI Average | BMI Average | BMI Average |
|  |  |  |  |  |  |
| Sugar and Processed Food Import (logged) | 0.063*** | 0.060*** | 0.065*** | 0.063*** | 0.061*** |
|  | (0.023) | (0.023) | (0.023) | (0.023) | (0.023) |
| KOF | 0.006*** |  |  |  |  |
|  | (0.002) |  |  |  |  |
| Economic Globalization |  | 0.004*** |  |  | 0.004*** |
|  |  | (0.001) |  |  | (0.001) |
| Political Globalization |  |  | 0.001 |  | 0.000 |
|  |  |  | (0.001) |  | (0.001) |
| Social Globalization |  |  |  | 0.003** | 0.002 |
|  |  |  |  | (0.002) | (0.002) |
| GDP capita (logged) | 0.709*** | 0.738*** | 0.772*** | 0.759*** | 0.718*** |
|  | (0.061) | (0.057) | (0.059) | (0.057) | (0.061) |
| ODA Aid (logged) | 0.002 | 0.002 | 0.001 | 0.001 | 0.002 |
|  | (0.001) | (0.001) | (0.001) | (0.001) | (0.001) |
| Health Expenditure per capita | 0.000*** | 0.000*** | 0.000*** | 0.000*** | 0.000*** |
|  | (0.000) | (0.000) | (0.000) | (0.000) | (0.000) |
| Population (logged) | 1.691*** | 1.725*** | 1.699*** | 1.714*** | 1.716*** |
|  | (0.110) | (0.109) | (0.111) | (0.110) | (0.111) |
| Age Dependency | -0.034*** | -0.034*** | -0.035*** | -0.035*** | -0.034*** |
|  | (0.002) | (0.002) | (0.002) | (0.002) | (0.002) |
| Total Import (logged) | -0.043** | -0.041** | -0.045** | -0.044** | -0.041** |
|  | (0.019) | (0.019) | (0.019) | (0.019) | (0.019) |
| Total Export (logged) | -0.003 | -0.003 | -0.003 | -0.003 | -0.003 |
|  | (0.005) | (0.005) | (0.005) | (0.005) | (0.005) |
| Democracy | -0.170*** | -0.181*** | -0.164*** | -0.165*** | -0.178*** |
|  | (0.057) | (0.057) | (0.057) | (0.057) | (0.057) |
| Interstate Conflict Severity | 0.000 | 0.000 | 0.000 | 0.000 | 0.000 |
|  | (0.000) | (0.000) | (0.000) | (0.000) | (0.000) |
| Intrastate Conflict | -0.057 | -0.075* | -0.064 | -0.071 | -0.069 |
|  | (0.045) | (0.044) | (0.046) | (0.044) | (0.046) |
| Constant | -5.080** | -5.763*** | -5.388** | -5.667*** | -5.559*** |
|  | (2.067) | (2.052) | (2.103) | (2.060) | (2.095) |
|  |  |  |  |  |  |
| Observations | 1,210 | 1,210 | 1,210 | 1,210 | 1,210 |
| R-squared | 0.815 | 0.816 | 0.814 | 0.814 | 0.816 |
| Number of Countries | 95 | 95 | 95 | 95 | 95 |

**Appendix F: Fiji Trade Agreement Profile**

| **Multilateral Agreements** | | |
| --- | --- | --- |
| 1993 | GATT | *General Agreement on Tariffs and Trade* |
| 1996 | WTO | *World Trade Organization Treaty* |
| 2007 | EPA | *Economic Partnership Association with the European Union*  EU provides duty and quota free access on all products except for sugar and rice; Fiji liberalised 84% of its imports from the EU over a period ending in 2023; improved sectoral rules of origin for canned fish & other goods |
| **Regional Agreements** | | |
| 1981 | SPARTECA | *South Pacific Regional Trade and Economic Cooperation Agreement*  Non-reciprocal trade agreement between 14 *FIC countries, Australia, & New Zealand; Duty free access to all products from FIC with the exception of sugar from Australia |
| 1998 | MSG | *Melanesian Spearhead Group*  Free trade agreement between Vanuatu, Papa New Guinea, Fiji, & Solomon Islands; elimination of tariffs for MSG goods; 2005 revision adopted ‘negative list approach’ to liberalisation of goods |
| 2001 | PICTA | *Pacific Island Countries Trade Agreement*  Signed in 2001, enforced in 2003; free trade for 14 FIC, Australia, & New Zealand; Fiji is required to reduce its maximum ad-varolem tariffs on PICTA originating goods to 15% in 2009, 10% by 2011, 5% by 2013 and zero by 2015 |
| 2001 | PACER | *Pacific Agreement on Closer Economic Relations*  Signed in 2001, enforced in 2002; Framework for facilitation of free trade and regional market for 14 FIC, Australia, & New Zealand |
| **Bilateral Agreements** | | |
| Current |  |  |
| 1999 | FATERA- Australia | *Fiji-Australia Trade and Economic Cooperation Agreement*  Framework for improving bilateral trade; no preferential arrangements |
| 2005 | China | Bilateral agreement on agriculture |
| Expired | | |
| USA 1994-2004; Non-reciprocal agreements with Vanuatu, Cook Islands, Papa New Guinea, & Tonga | | |

*FIC- Forum Island Countries

Source: World Trade Organisation, Trade Policy Review, 2009

World Trade Organisation, 2016
